# COVID-19 patient with coronary thrombosis supported with ECMO and Impella 5.0 ventricular assist device: a case report

**DOI:** 10.1093/ehjcr/ytaa342

**Published:** 2020-11-20

**Authors:** Kamen Valchanov, Unni Krishnan, Stephen P Hoole, Will R Davies, Stephen Pettit, Nicola Jones, Jas Parmar, Pedro Catarino, Mohamed Osman, Marius Berman

**Affiliations:** Royal Papworth Hospital, Cambridge CB2 0AY, UK

**Keywords:** Case report, COVID-19, Coronary thrombosis, ECMO, Impella

## Abstract

**Background:**

COVID-19 can present with cardiovascular complications.

**Case summary:**

We present a case report of a 43-year-old previously fit patient who suffered from severe acute respiratory syndrome coronavirus 2 (SARS-CoV-2) infection with thrombosis of the coronary arteries causing acute myocardial infarction. These were treated with coronary stenting during which the patient suffered cardiac arrest. He was supported with automated chest compressions followed by peripheral veno-arterial extracorporeal membrane oxygenation (VA ECMO). No immediate recovery of the myocardial function was observed and, after insufficient venting of the left ventricle was diagnosed, an Impella 5 pump was implanted. The cardiovascular function recovered sufficiently and ECMO was explanted and inotropic infusions discontinued. Due to SARS-CoV-2 pulmonary infection, hypoxia became resistant to conventional mechanical ventilation and the patient was nursed prone overnight. After initial recovery of respiratory function, the patient received a tracheostomy and was allowed to wake up. Following a short period of agitation his neurological function recovered completely. During the third week of recovery, progressive multisystem dysfunction, possibly related to COVID-19, developed into multiorgan failure, and the patient died.

**Discussion:**

We believe that this is the first case report of coronary thrombosis related to COVID-19. Despite the negative outcome in this patient, we suggest that complex patients may in the future benefit from advanced cardiovascular support, and may even be nursed safely in the prone position with Impella devices.

**Learning points**

- Myocardial injury is not uncommon in patients with COVID-19. It can be combined with pulmonary dysfunction. In this case, coronary thrombosis was the leading presentation.
- Cardiac arrest management with advanced mechanical circulatory support for COVID-19 patients is challenging in an area of isolation. A multidisciplinary team is best to select and manage the optimal device.
- Respiratory and multiorgan failure are most problematic in severe myocardial dysfunction related to COVID-19. Impella-supported patients can safely be nursed in the prone position to improve oxygenation.

## Timeline

**Table:** 

Day 1	Patient presents to local hospital with pyrexia and chest pain
	Patient transferred to cardiac centre
	Coronary angiography showing thrombosis
	Cardiac arrest and ECMO support
	Coronary stenting after resuscitation
	BAL samples negative for COVID-19
Day 2	Severe LV dysfunction not improving with inotropic infusions
Day 3	Impella 5 inserted via left subclavian artery
Day 4	Cardiac index 3.2 L/min/m^2^, CVP 8, and PCWP 13
Day 6	BAL sample positive for COVID-19
	CT showing worsening ground-glass opacification of both lungs
Day 8	Nursed in the prone position to improve hypoxaemia
Day 9	Hypoxia improved and tracheostomy inserted
Day 14	Worsening renal function and haemofiltration
Day 16	Liver dysfunction and melaena
Day 20	Worsening hypoxia and prone positioning
Day 21	Refractory multiorgan failure and death

## Other specialities involved

Anaesthesia, Intensive care, Cardiology, Surgery

## Introduction

COVID-19 caused by severe acute respiratory syndrom coronavirus 2 (SARS-CoV-2) infection can present with cardiovascular complications. Early on in the COVID-19 pandemic, several publications referred to different cardiac complications. The exact mechanisms of how SARS-CoV-2 can cause myocardial injury are not clearly understood. The proposed mechanisms of myocardial injury are direct damage to the cardiomyocytes, systemic inflammation, myocardial interstitial fibrosis, interferon-mediated immune response, exaggerated cytokine response by Type 1 and 2 helper T cells, in addition to coronary plaque destabilization and hypoxia.^1^ Coronary thrombosis has not been previously reported in the literature.

A 43-year-old male smoker, with no prior medical history or current medication, presented to his local hospital with severe central chest pain on the background of 3 days of intermittent chest discomfort and pyrexia. A 12-lead ECG showed global ischaemic changes, including ST-segment elevation in aVR and concurrent ST-segment depression in the inferolateral leads. He was transferred emergently to our tertiary cardiac centre after administration of loading doses of antiplatelet therapy (aspirin 600 mg and clopidogrel 600 mg per oral stat). On arrival, he was haemodynamically stable (blood pressure 120/99 mmHg and pulse rate 149) but hypoxic (saturations below 90% on air), requiring high flow oxygen albeit without clinical evidence of pulmonary oedema. Physical examination was unremarkable except cyanosis on air. Bedside transthoracic echocardiography (TTE) was consistent with an extensive left anterior descending (LAD) artery territory myocardial infarction (MI) with moderate left ventricular (LV) systolic dysfunction, normal valvular architecture and function, and no pericardial collection.

**Figure 1:**
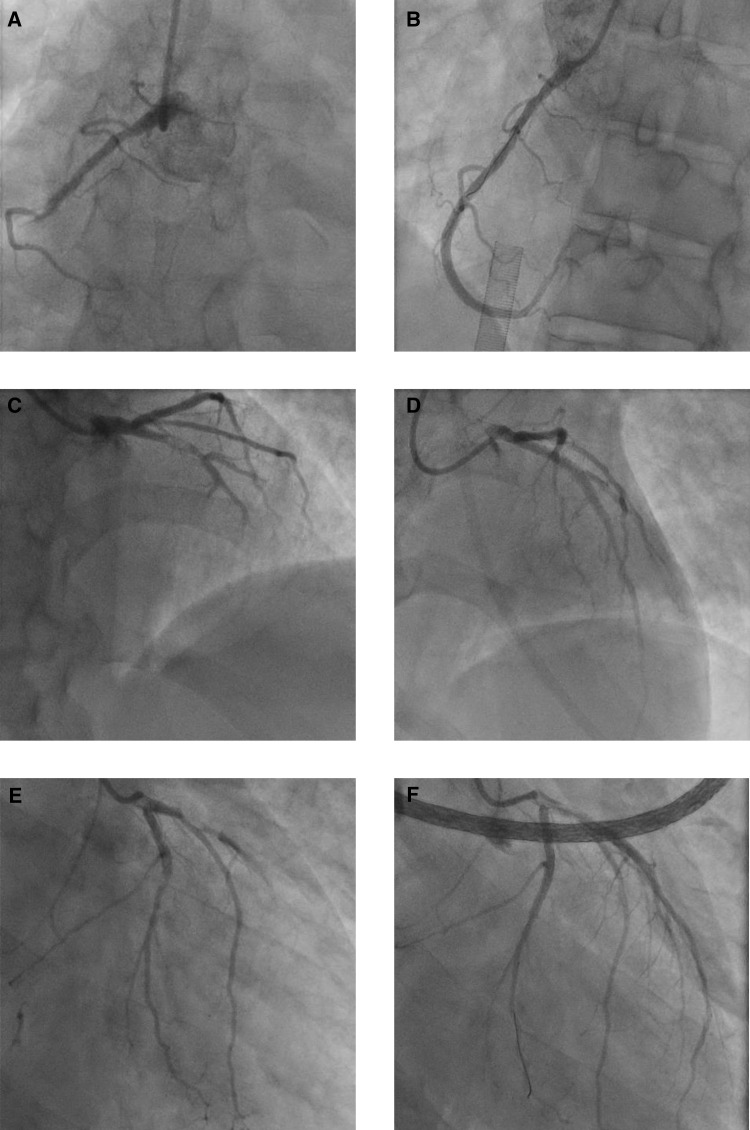
Coronary angiography imaging at presentation.

Coronary angiography demonstrated a dominant right coronary artery (RCA) occluded in the mid segment (*Figure 1A*;Supplementary material online, *Video S1*), a normal left main coronary artery, a proximal segment occlusion of the LAD with thrombus *in situ* (*Figure 1B*;Supplementary material online, *Video S2*). and a non-dominant left circumflex artery (LCx) with a severe mid segment lesion (*Figure 1C*;Supplementary material online, *Video S3*). Following systemic heparinization (5000 IU bolus followed by an infusion) and concurrent administration of a glycoprotein 2-beta 3-alpha inhibitor (GP2b3ai), tirofiban, 400 ng/kg/min for 30 min to counter the significant thrombotic burden, a 3.5 mm × 33 mm drug-eluting stent (DES) was deployed in the LAD. The patient then developed persistent ventricular tachycardia (VT) deteriorating to a PEA (pulseless electrical activity) cardiac arrest with a narrow complex tachycardia following defibrillation. Cardiopulmonary resuscitation was promptly initiated including endotracheal intubation and automated chest compressions until veno-arterial extracorporeal membrane oxygenation (VA ECMO) could be started (20 min). Vascular cannulae were placed in the left femoral artery (19 F Maquet arterial cannula) and right femoral vein (25/55 F Maquet cannula), and VA ECMO was established. A repeat TTE was performed and there was no evidence of cardiac mechanical complications such as ventricular septal rupture, papillary muscle rupture, or pericardial tamponade. We then proceeded to treat the RCA lesion with two overlapping DES (3.5 mm × 33 mm and 3.5 mm × 38 mm) and the LCx lesion with a 3.0 mm × 18 mm DES, achieving a good angiographic result and TIMI 3 flow in all three vessels (*Figure 1D–F*).

**Figure 2:**
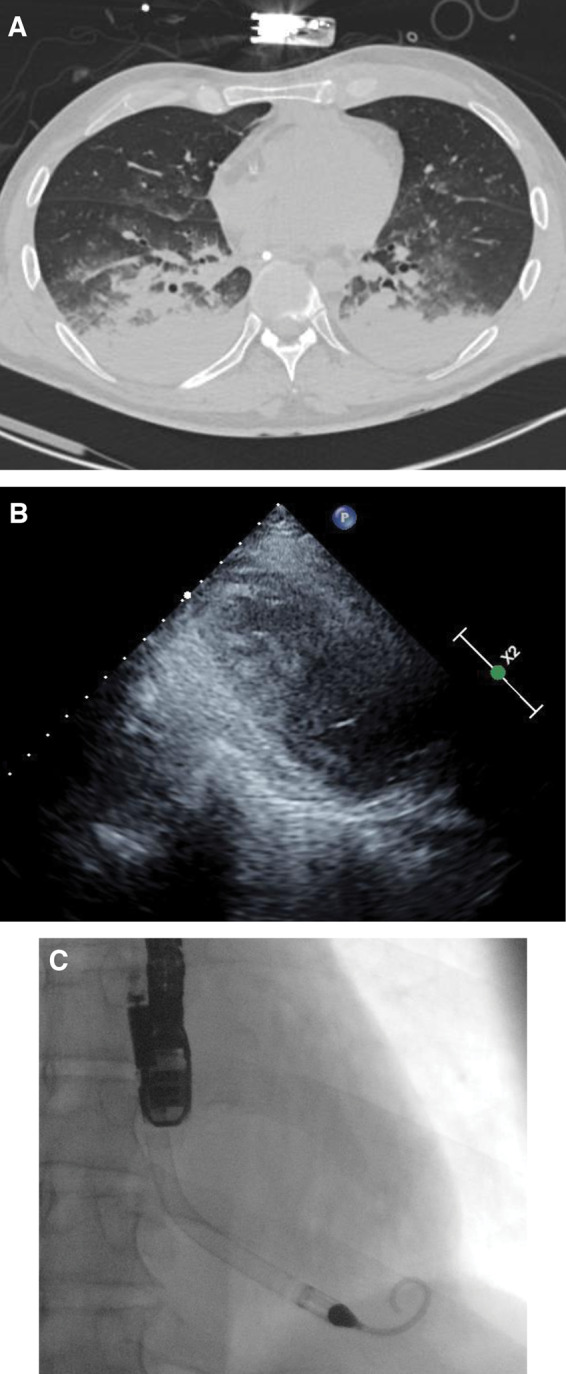
(*A*) CT chest consistent with interstitial lung infection. (*B*) Trans-thoracic echocardiograph with spontaneous echo contrast, on peripheral VA ECMO, prior to Impella implant. (*C*) Trans-oesophageal confirmation of Impella position.

There was no immediate improvement in LV function despite satisfactory revascularization. With VA ECMO flow of 4.5 L/min, the mean arterial pressure was 75 mmHg and the pulse pressure was 20 mmHg, suggesting that the left ventricle was struggling to eject. Infusions of dopamine 5 μg/kg/min and adrenaline 0.02 μg/kg/min were started in an attempt to maintain LV ejection and reduce the risk of LV distension and thrombosis. A computed tomography (CT) scan of chest, abdomen, and pelvis was performed before transfer to the intensive care unit (ICU). This demonstrated extensive consolidation in the dependent portion of the lungs (*Figure 2A*), as well as mild interlobular septal thickening and centrilobular ground-glass change anterior to the consolidation, and ruled out other thrombotic areas. A distended left ventricle was also noted, with mild apical para-septal emphysema, and multiple rib fractures attributed to automated chest compressions.

Blood results on arrival in the ICU (*Table 1*) were significantly deranged including a haemoglobin 145 g/L (reference range 135–172), white cell count 25.9 × 10^9^/L (reference range 3.9–10.2), neutrophil count 22.66 × 10^9^/L (reference range 1.5–7), lymphocyte count 1.48 × 10^9^/L (reference range 1.1–4.5), ferritin 1430.9 μg/L (reference range 22–322), C-reactive protein 167 mg/L (reference range 0–6), high sensitivity troponin-I 18 509 ng/L (reference range 0–58), D-dimer 2296 ng/L (reference range 0–230), interleukin-6 (IL-6) 511 pg/mL (reference range 0–2), and tumour necrosis factor (TNF) alpha 13.89 pg/mL (reference range 0–5). Naso-pharyngeal swabs were sent for COVID-19 RNA analysis. Broad-spectrum antibiotic therapy with piperacillin–tazobactam was started. Azithromycin 500 mg once a day was added because there was clinical suspicion of underlying SARS-CoV-2 infection, given persistent pyrexia, unexpected hypoxia at the time of initial presentation, and blood tests compatible with COVID-19.

**Table 1:** Blood tests results on admission, after 24 h, and after 14 days

Parameter (unit)	On admission	After 24 h	After 14 days	Reference range
Haemoglobin (g/L)	145	79	88	135–172
Mean corpuscular volume (fL)	84.9	82.9	86.3	80.0–99.0
White cell count (×10^9^/L)	25.9	19.7	33.7	3.9–10.2
Neutrophils (×10^9^/L)	22.66	16.43	29.29	1.5–7.0
Lymphocytes (×10^9^/L)	1.48	1.69	1.85	1.1–4.5
Neutrophil/lymphocyte ratio	15.5	9.7	15.7	<3.0
Eosinophil count (×10^9^/L)	0.03	0.02	0.07	0.02–0.5
Platelet count (×10^9^/L)	338	170	261	150–370
Creatinine (μmol/L)	116	106	109	62–115
Alanine aminotransferase (U/L)	84	330	1088	10–49
Alkaline phosphatase (U/L)	87	44	110	30–130
High sensitivity troponin (ng/L)	18 509.6	>25 000	6871.2	0.0–0.58
C-reactive protein (mg/L)	167	259	96	0–6
Procalcitonin (ng/mL)	1.81		5.29	0.0–5.0
Ferritin (μg/L)	1430.9		9547.8	22.0–322
D-dimer(ng/mL)	2296		3197	0–230
Interferon-γ (pg/mL)	<0.94			<10.00
Interleukin-1β (pg/mL)	1.74			0.0–3.1
Interleukin-10 (pg/mL)	6.13			0.0–1.0
Interleukin-6 (pg/mL)	511.02			0.0–2.0
Tumour necrosis factor-α (pg/mL)	13.89			0.0–5.0

Repeat TTE at 24 h after revascularization demonstrated a mildly dilated left ventricle with severe LV systolic dysfunction (ejection fraction <5%) and spontaneous echo contrast in the LV cavity (*Figure 2B*), despite the ongoing use of two inotropes.

In order to unload the left ventricle, the shock team decided to place an Impella 5.0 (Abiomed, Danvers, MA, USA), which was inserted on Day 3 via a 10 mm graft on the left subclavian artery as a bridge to decision (*Figure 2C*).^2^ Position was confirmed by trans-oesophageal echocardiography (TEE) and fluoroscopy. We were able to achieve flow of 4.5 L/min on P8. Peripheral VA ECMO flow was gradually reduced from 4 L/min to 2 L/min over the next 24 h, after which the peripheral vascular cannulae were removed. At this stage, the patient’s haemodynamic function was supported with an Impella alone. The pulmonary artery flotation catheter (PAFC) indicated a cardiac index of 3.2 L/min/m^2^, with a central venous pressure of 8 mmHg and a pulmonary capillary wedge pressure of 13 mmHg.

**Figure 3:**
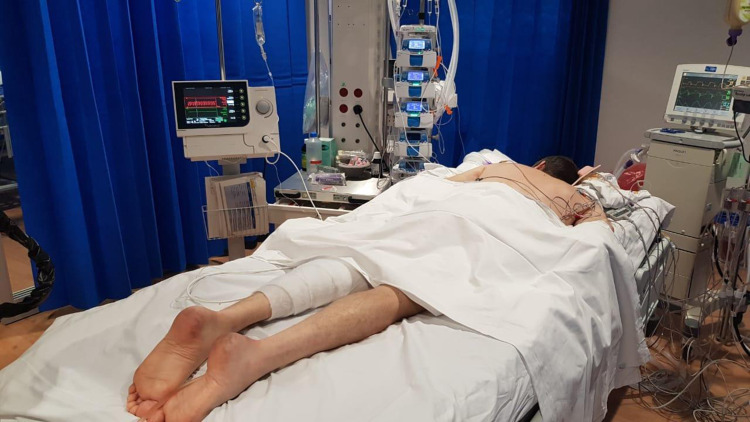
The patient in the prone position with the Impella 5.0 placed via a 10 mm graft in the left axillary artery. The drive line is running alongside the body, secured to his left calf, avoiding loops.

The patient was barrier nursed because of clinical suspicion of COVID-19. However, three naso-pharyngeal swabs were negative for SARS-CoV-2 RNA. BAL (broncho-alveolar lavage) sample on arrival in the ICU was also negative. On Day 6, bronchoscopy was required to clear secretions and the second BAL sample was sent for analysis. This was positive for SARS-CoV-2 RNA. The patient developed new bilateral consolidation on a chest radiograph despite effective mechanical unloading of the left ventricle. Haemodynamics remained stable during this time, with regular echocardiography confirming satisfactory Impella position, adequate LV venting, and good right ventricular function. A repeat chest CT showed worsening ground-glass opacification of both lungs. There was progressive deterioration in gas exchange. On Day 8, the patient was proned for 24 h, which resulted in significant improvement in gas exchange (*Figure 3*).

Tracheostomy was performed on Day 9 in order to reduce sedation and allow neurological assessment. The patient woke up within 48 h and was interacting with staff with a Glasgow Coma Score (GCS) of 14. Unfortunately, there was progressive end-organ dysfunction from Day 14. The patient became oliguric and the serum creatinine rose to 109 μmol/L. Continuous veno-venous haemofiltration was commenced. There was biochemical evidence of acute liver injury, with alanine aminotransferase (ALT) 1088 IU/L, bilirubin 22 μmol/L, and alkaline phosphatase (AP) 110 U/L. There was evidence of gastrointestinal bleeding with episodes of melaena. The general condition of the patient worsened over the following week, with hypotension requiring vasopressor support and a worsening neurological state. Gas exchange deteriorated and he developed refractory hypoxia despite mechanical ventilation, use of muscle relaxation, and periods of prone ventilation. Liver function tests continued to deteriorate, and he required treatment for hypoglycaemia. Ultimately, he developed a refractory metabolic acidosis despite haemofiltration at an exchange rate of 35 ml/kg/h. The Impella 5.0 continued to provide excellent cardiac support during this time, with a cardiac index (CI) in excess of 3 L/min/m^2^.

Our multidisciplinary team recognized that ongoing support was not in the patient’s best interests, given severe and progressive multiorgan failure despite maximal treatment. End of life care was instituted, and the patient sadly passed away.

## Discussion

We present the case of a young patient who presented with atypical symptoms of COVID-19. He had no medical history other than being a current smoker. His body mass index was within the normal range. A minor plaque in the LAD was noted. However, we propose that the multiple coronary occlusions may be precipitated by the procoagulant status secondary to COVID-19.^3^ Aggressive antithrombotic therapy and prompt coronary revascularization did not result in recovery of myocardial function and the patient required mechanical circulatory support.

The VA ECMO support was not a durable option, and venting of the left ventricle was problematic. Failure to unload the left ventricle in this situation is associated with a high risk of LV thrombosis and death. The shock team frequently reviewed the clinical situation and agreed that Impella 5 was the best therapy strategy. The unusual treatment in our case is that the patient needed nursing in the prone position to improve oxygenation. This is the first case, to our knowledge, when an Impella-supported patient who has been proned. This did not result in malposition of the device and the function was maintained throughout.

A similar case where the presenting feature of COVID was myocarditis and pericardial effusion has been reported.^4^ However, in this case, there was no coronary thrombosis. There are emerging reports of COVID-19-related coronary pathologies, such as spontaneous dissection.^5^ It is possible that in our case a minor atheromatous plaque and current smoking status provided the substrate for catastrophic coronary thrombosis in the setting of severe COVID-19. From the earlier case series, it is clear that SARS-CoV-2 infection has myocardial complications in patients with no previously known cardiovascular disease, with the incidence being as high as 13.2%.^6^ From the New York case series, it is known that acute myocardial infarction occurs in COVID-19 patients,^5^ but in our case it also led to coronary thrombosis, and subsequently the patient suffered cardiac arrest.^7^

The first BAL results for COVID-19 were negative. Despite the atypical symptoms on presentation, the blood tests and chest CT images, as well as clinical evolution were highly suspicious for this infection. The patient was nursed in isolation throughout. The second BAL results returned positive, which raises a suspicion of nosocomial SARS-CoV-2 infection, but we feel that the clinical picture supported by lung imaging and blood results indicates primary infection at the time of presentation.

There were several important learning points for the shock team. Effective communication, team working, and decision-making in the catheterization laboratory are more challenging when operators are using personal protective equipment. There may be diagnostic uncertainty when initial nasopharyngeal swabs for COVID-19 RNA are negative in a patient with a high pre-test probability for COVID-19. It is important to minimize the risk of nosocomial transmission of COVID-19. Finally, patients may succumb to fulminant COVID-19 despite effective mechanical circulatory support. Shock teams should select temporary mechanical circulatory support devices in the initial stages of illness and should be prepared for the possibility of adverse outcomes.

## Conclusion

COVID-19 can present with cardiovascular complications including coronary thrombosis. These complex patients can be effectively supported with VA-ECMO and Impella 5, and can be safely nursed in the prone position.

## Lead author biography

**Figure:**
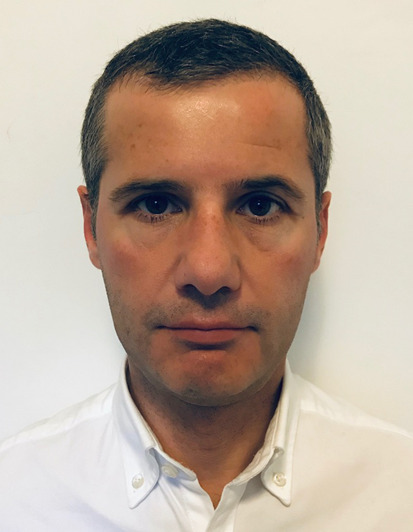


Dr Kamen Valchanov has been a Consultant in Anaesthesia and Intensive Care in the Royal Papworth Hospital, Cambridge, UK since 2007. His main research interests are ECMO, mechanical cardiovascular support, and sedation and analgesia for critically ill patients. He also specialzes in anaesthesia for pulmonary hypertensive surgery (pulmonary endarterectomy) and complex aortic surgery. In addition to this, he runs a chronic pain clinic which provides spinal cord stimulation for refractory angina pectoris patients.

## Supplementary material

Supplementary material are available at *European Heart Journal - Case Reports* online.

## Supplementary Material

ytaa342_Supplementary_Data

## References

[1] Farrokhan-Babapoor S, Gill D, Walker J, Rasekhi RT, Bozorgnia B, Amanullah A. Myocardial injury and COVID-19; possible mechanisms. Life Sci 2020:253;117723 32360126 10.1016/j.lfs.2020.117723 PMC7194533

[2] Tehrani BN, Truesdell AG, Sherwood MW, Desai S, Tran HA, Epps KC, Singh R, Psotka M, Shah P, Cooper LB, Rosner C,, Raja A, Barnett SD, Saulino P, deFilippi CR, Gurbel PA, Murphy CE, O’Connor CM. Standardized team-based care for cardiogenic shock. J Am Coll Cardiol 2019;73:1659–1669. 30947919 10.1016/j.jacc.2018.12.084

[3] Ranucci M, Ballotta A, Di Dedda U, Bayshnikova E, Dei Poli M, Resta M, Falco M, Albano G, Menicanti L. The procoagulant pattern of patients with COVID-19 acute respiratory distress syndrome. J Thromb Haemosts 2020;18:1747–1751 10.1111/jth.14854 PMC9906332 32302448

[4] Inciardi RM, Lupi L, Zaccone G, Italia L, Raffo M, Tomasoni D, Cani DS, Cerini M, Farina D, Gavazzi E, Maroldi R, Adamo M, Ammirati E, Sinagra G, Lombardi CM, Metra M. Cardiac involvement in a patient with coronavirus disease 2019 (COVID-19). JAMA Cardiol 2020;5:1–6. 10.1001/jamacardio.2020.1096 PMC7364333 32219357

[5] Kumar K, Vogt JC, Divanji PH, Cigarroa JE. Spontaneous coronary artery dissection of the left anterior descending artery in a patient with COVID-19. Catheter Cardiovasc Interv 2020:doi: 10.1002/ccd.28960. 10.1002/ccd.28960 PMC7267179 32383284

[6] Guo T, Fan Y, Chen M, Wu X, Zhang L, He T, Wang H, Wan J, Wang X, Lu Z. Cardiovascular implications of fatal outcomes of patients with coronavirus disease 2019 (COVID-19). JAMA Cardiol 2020;5:1–8. 10.1001/jamacardio.2020.1017 PMC7101506 32219356

[7] Bangalore S, Sharma A, Slotwiner A, Yatskar L, Harari R, Shah B, Ibrahim H, Friedman GH, Thompson C, Alviar CL, Chadow HL, Fishman GI, Reynolds HR, Keller N, Hochman JS. ST-segment elevation in patients with Covid-19 – a case series. N Engl J Med 2020;382:2478–2480. 32302081 10.1056/NEJMc2009020 PMC7182015

