# Supplementary material for: COVID-19 patient with coronary thrombosis supported with ECMO and Impella 5.0 ventricular assist device: a case report
Source: Eur Heart J Case Rep. 2020 Nov 20;4(6):1–6. doi: 10.1093/ehjcr/ytaa342 (PMC7717226; doi:10.1093/ehjcr/ytaa342)
Supplement: ytaa342_Supplementary_Data [file ytaa342_supplementary_data.zip › ytaa342-suppl_data/EHJ-CR-Slide-Set COVID-19 Revised.pptx]

## Slide 1
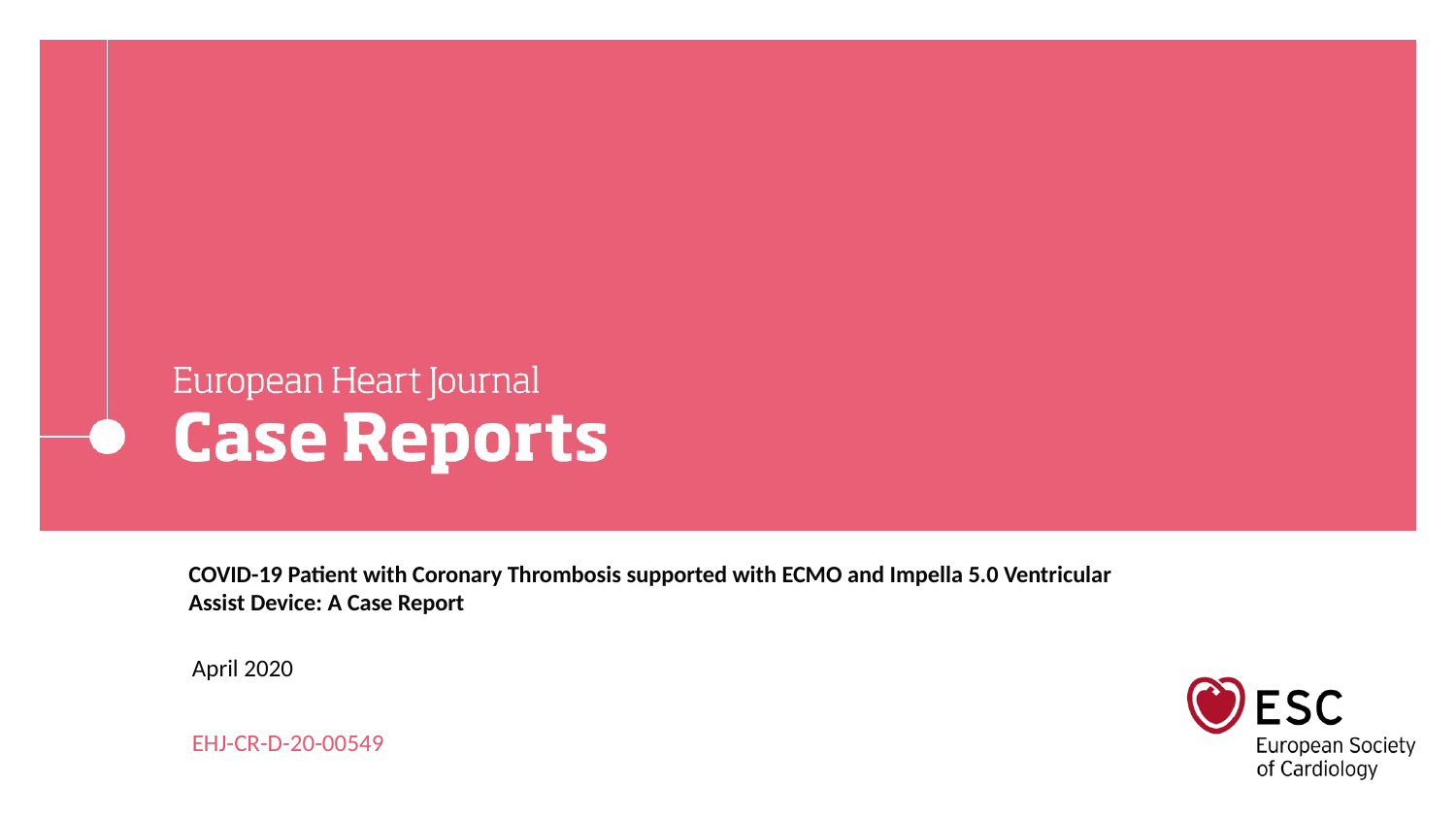

# COVID-19 Patient with Coronary Thrombosis supported with ECMO and Impella 5.0 Ventricular Assist Device: A Case Report
April 2020
EHJ-CR-D-20-00549

## Slide 2
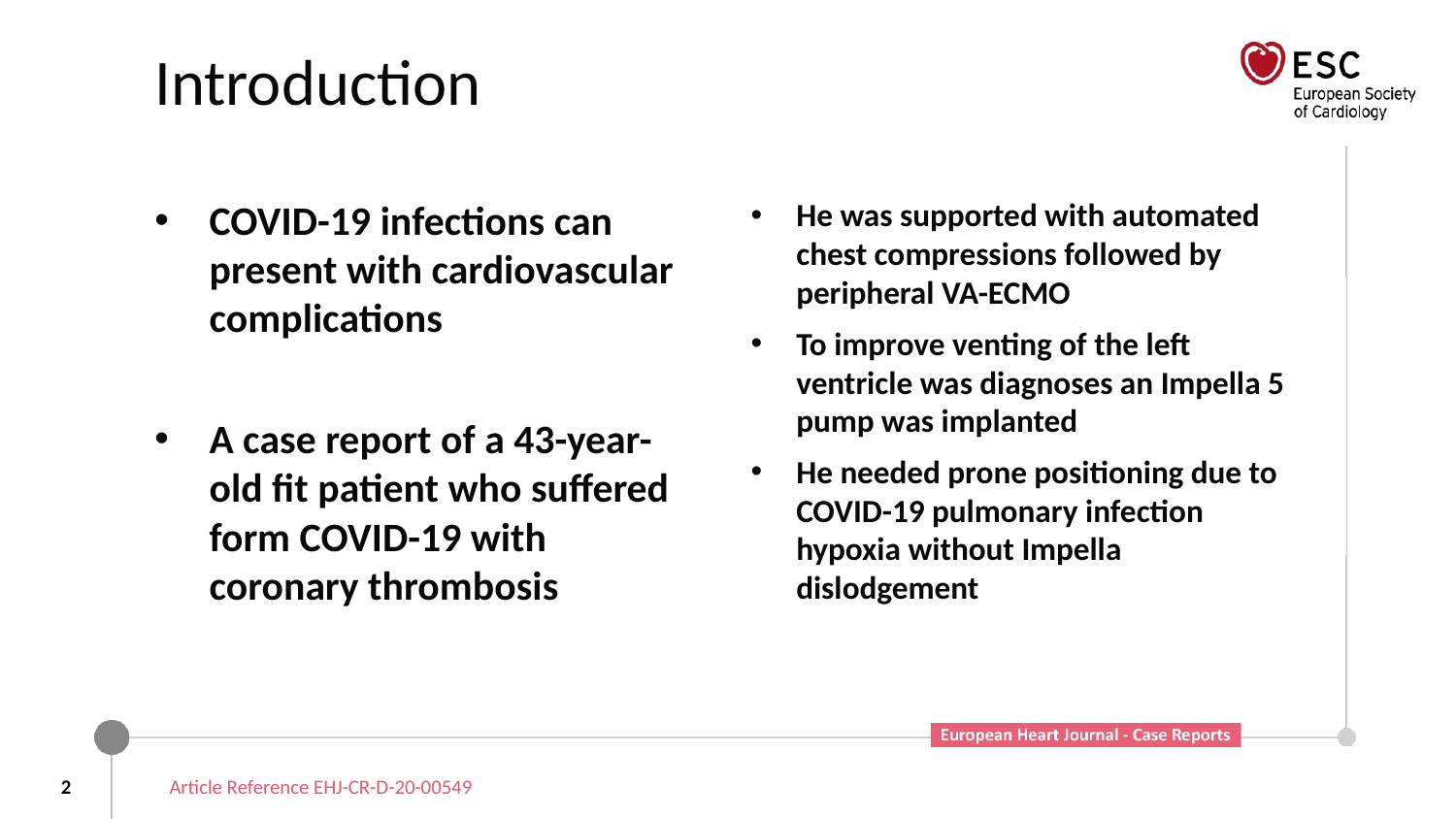

# Introduction
COVID-19 infections can present with cardiovascular complications
A case report of a 43-year-old fit patient who suffered form COVID-19 with coronary thrombosis
He was supported with automated chest compressions followed by peripheral VA-ECMO
To improve venting of the left ventricle was diagnoses an Impella 5 pump was implanted
He needed prone positioning due to COVID-19 pulmonary infection hypoxia without Impella dislodgement
2
Article Reference EHJ-CR-D-20-00549

## Slide 3
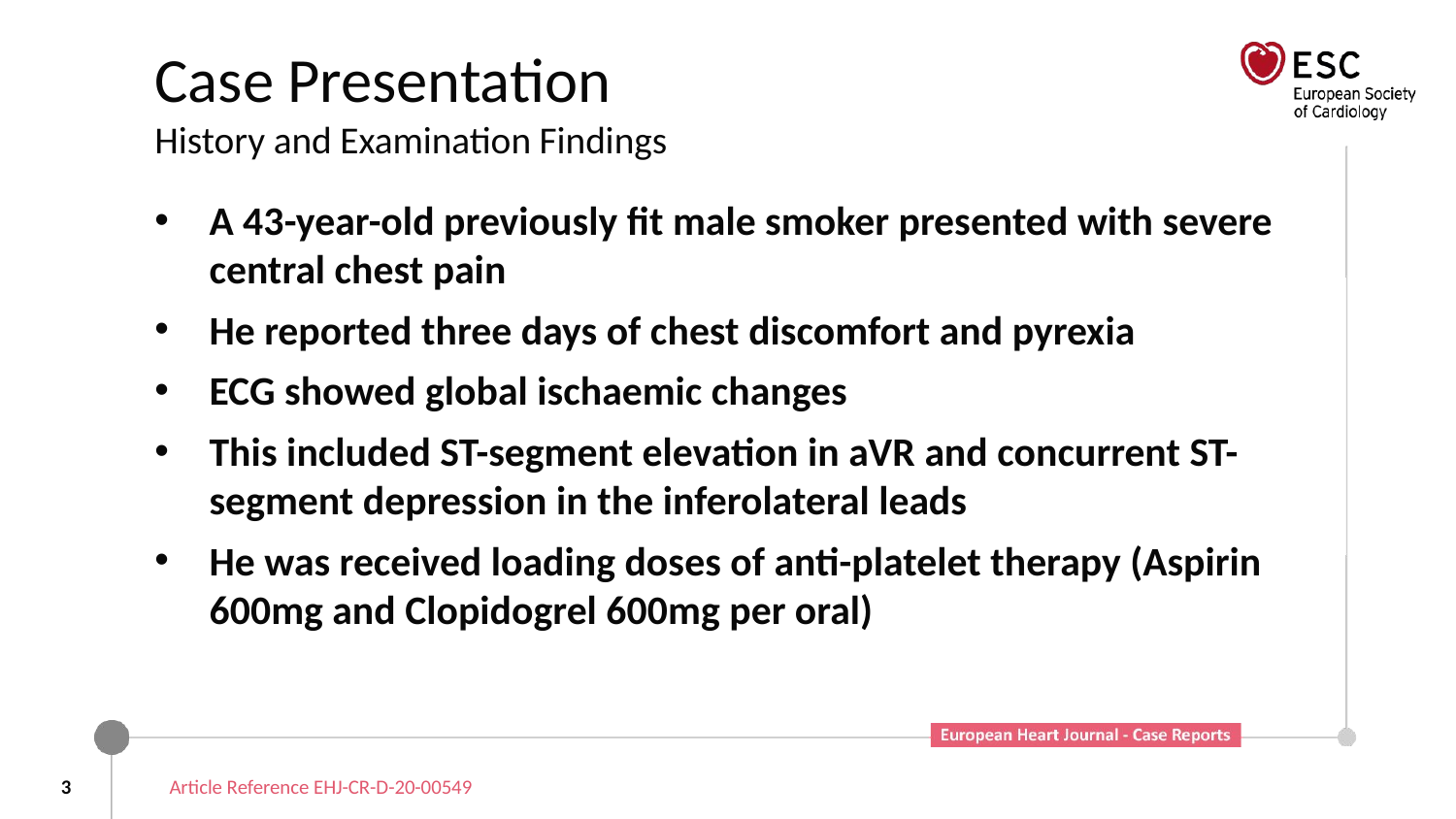

# Case Presentation History and Examination Findings
A 43-year-old previously fit male smoker presented with severe central chest pain
He reported three days of chest discomfort and pyrexia
ECG showed global ischaemic changes
This included ST-segment elevation in aVR and concurrent ST-segment depression in the inferolateral leads
He was received loading doses of anti-platelet therapy (Aspirin 600mg and Clopidogrel 600mg per oral)
3
Article Reference EHJ-CR-D-20-00549

## Slide 4
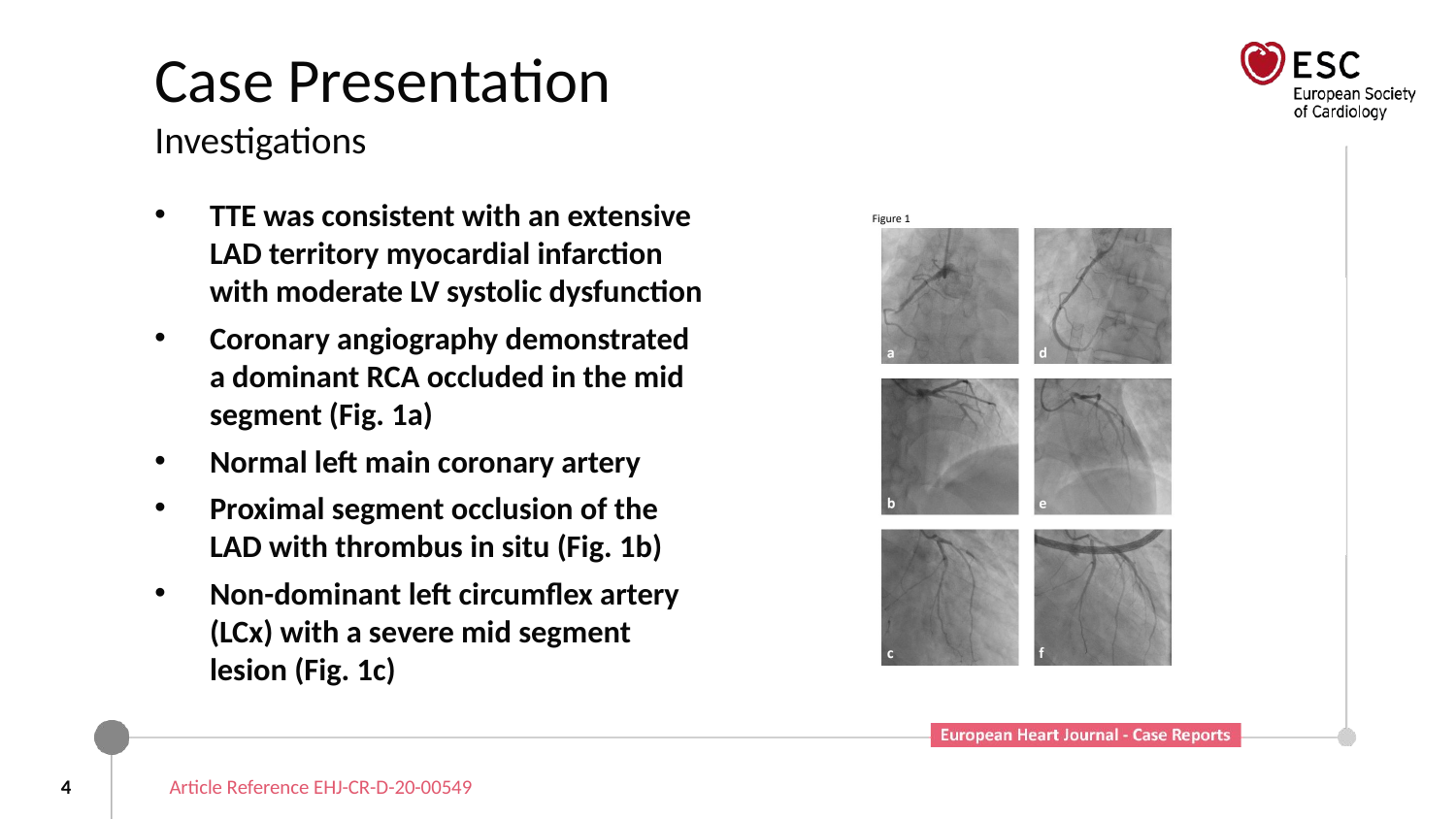

# Case Presentation Investigations
TTE was consistent with an extensive LAD territory myocardial infarction with moderate LV systolic dysfunction
Coronary angiography demonstrated a dominant RCA occluded in the mid segment (Fig. 1a)
Normal left main coronary artery
Proximal segment occlusion of the LAD with thrombus in situ (Fig. 1b)
Non-dominant left circumflex artery (LCx) with a severe mid segment lesion (Fig. 1c)
4
Article Reference EHJ-CR-D-20-00549

## Slide 5
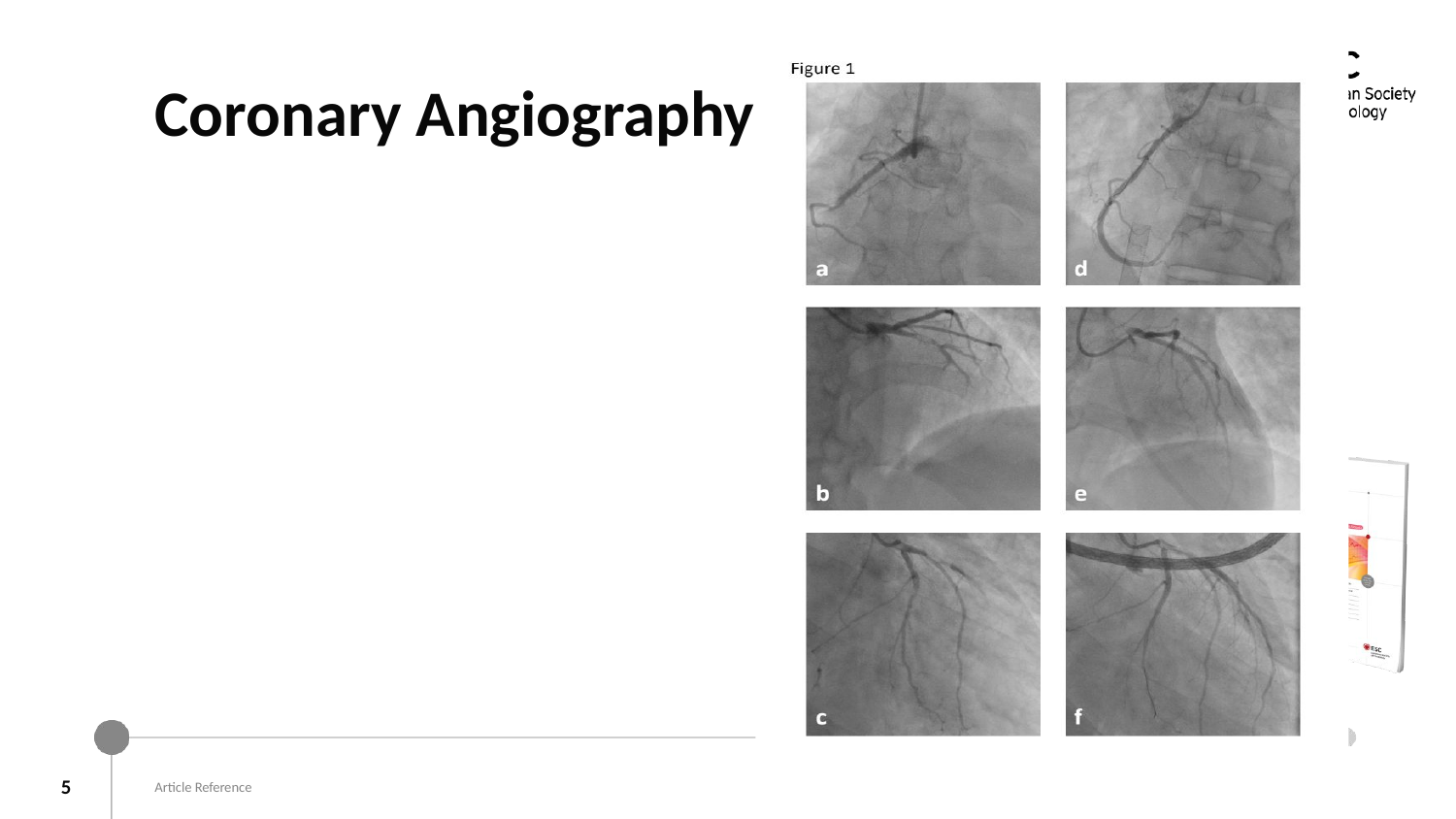

# Coronary Angiography
5
Article Reference

## Slide 6
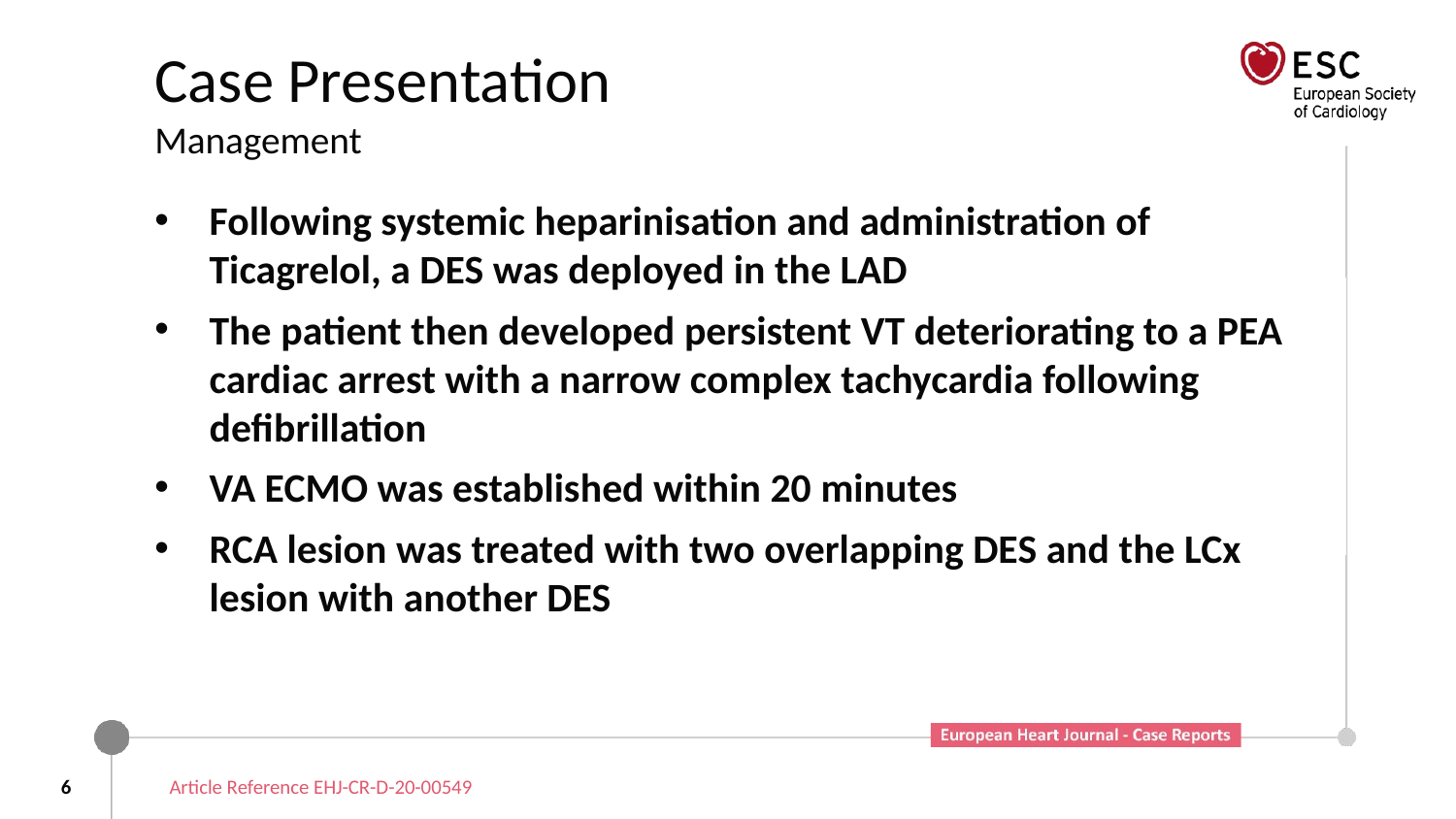

# Case Presentation Management
Following systemic heparinisation and administration of Ticagrelol, a DES was deployed in the LAD
The patient then developed persistent VT deteriorating to a PEA cardiac arrest with a narrow complex tachycardia following defibrillation
VA ECMO was established within 20 minutes
RCA lesion was treated with two overlapping DES and the LCx lesion with another DES
6
Article Reference EHJ-CR-D-20-00549

## Slide 7
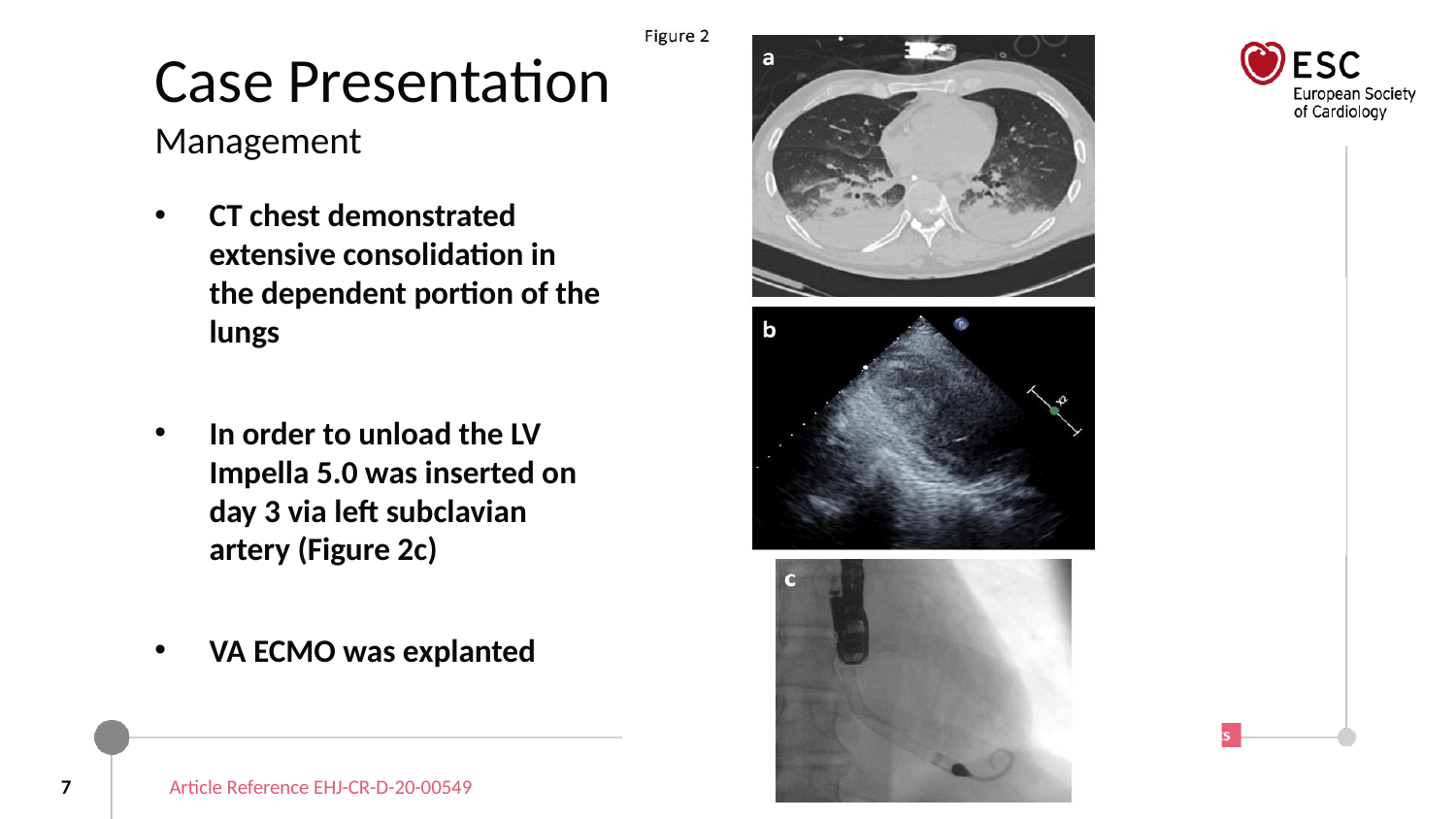

# Case Presentation Management
CT chest demonstrated extensive consolidation in the dependent portion of the lungs
In order to unload the LV Impella 5.0 was inserted on day 3 via left subclavian artery (Figure 2c)
VA ECMO was explanted
7
Article Reference EHJ-CR-D-20-00549

## Slide 8
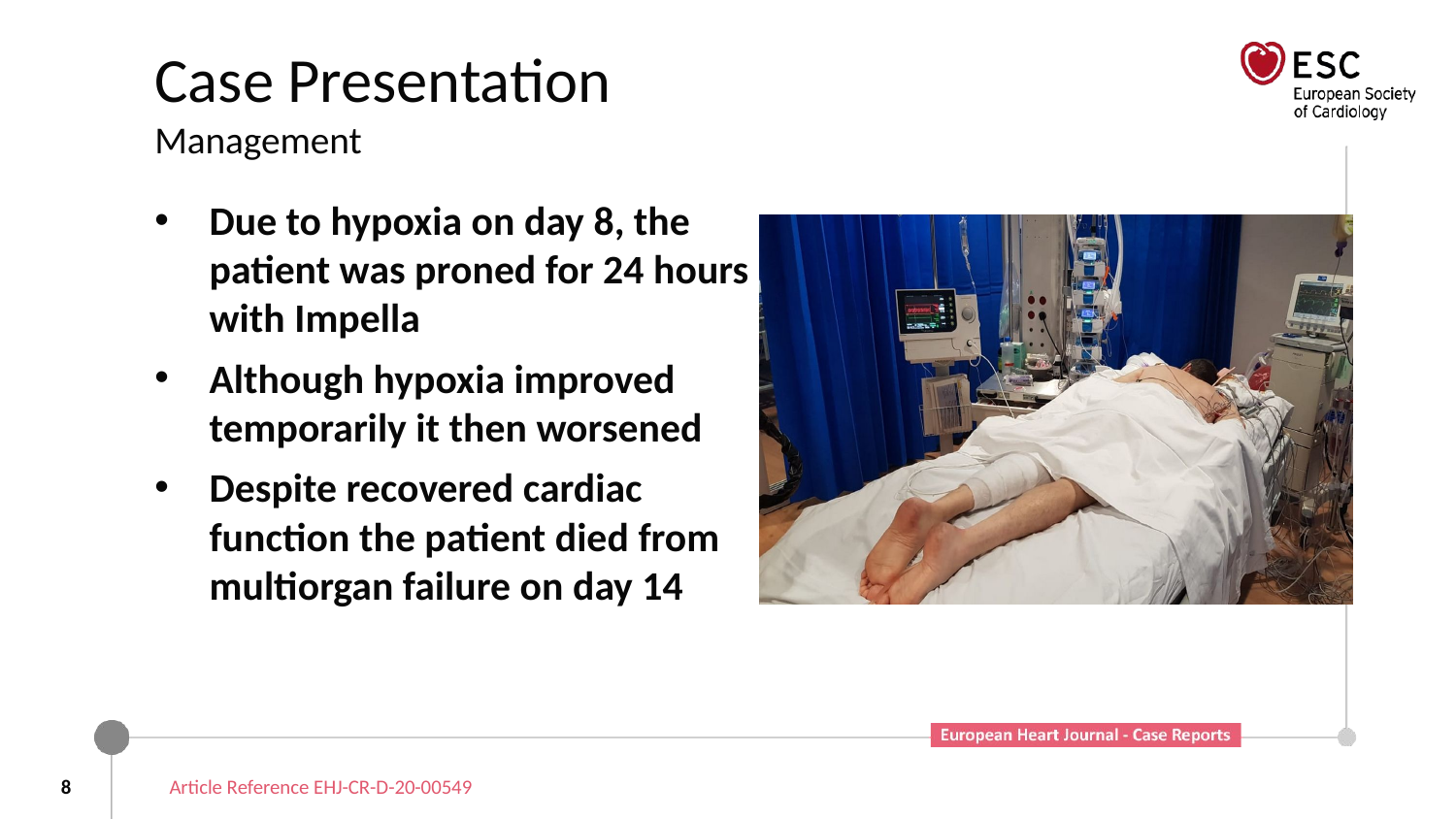

# Case Presentation Management
Due to hypoxia on day 8, the patient was proned for 24 hours with Impella
Although hypoxia improved temporarily it then worsened
Despite recovered cardiac function the patient died from multiorgan failure on day 14
8
Article Reference EHJ-CR-D-20-00549

## Slide 9
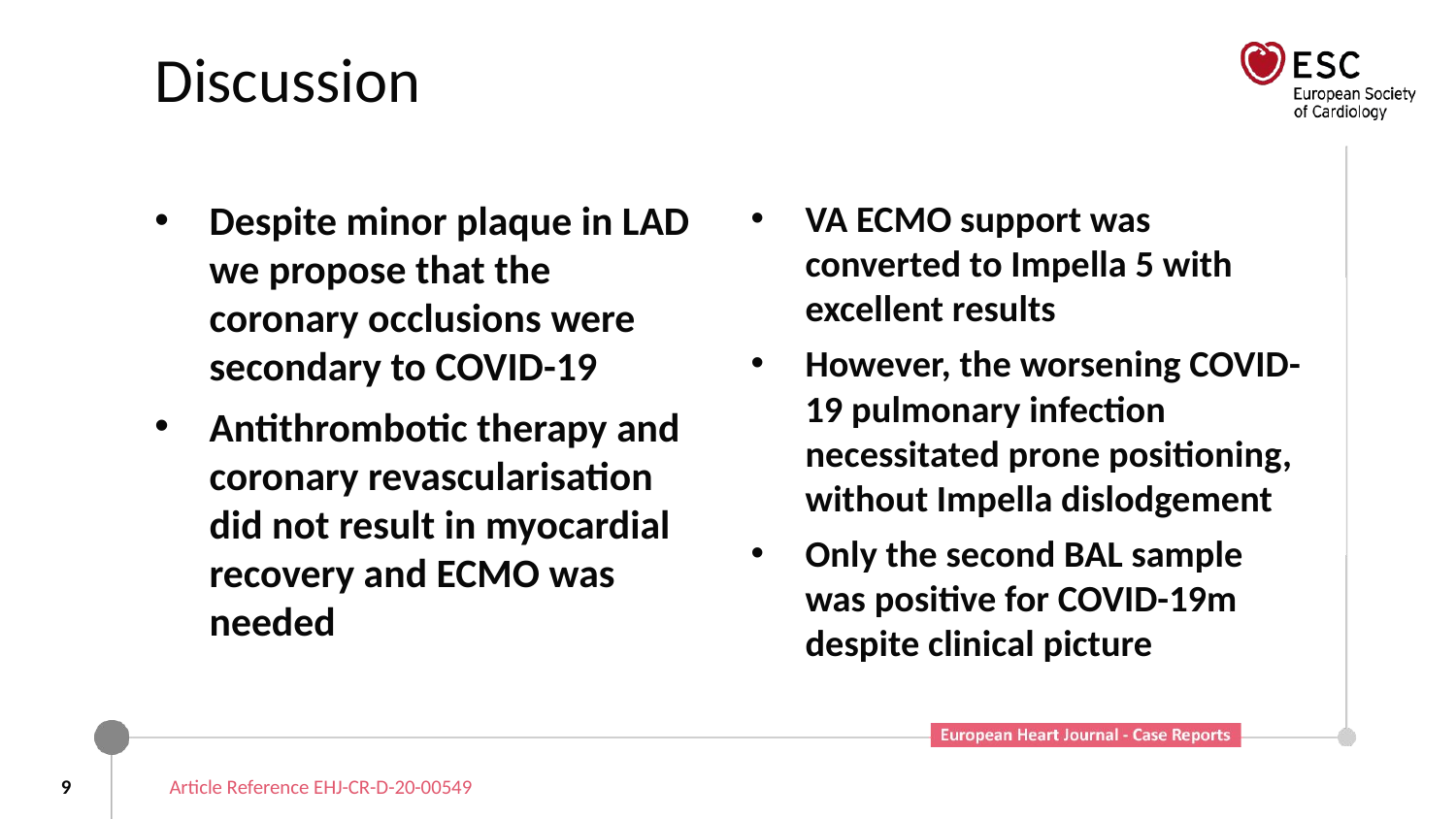

# Discussion
Despite minor plaque in LAD we propose that the coronary occlusions were secondary to COVID-19
Antithrombotic therapy and coronary revascularisation did not result in myocardial recovery and ECMO was needed
VA ECMO support was converted to Impella 5 with excellent results
However, the worsening COVID-19 pulmonary infection necessitated prone positioning, without Impella dislodgement
Only the second BAL sample was positive for COVID-19m despite clinical picture
9
Article Reference EHJ-CR-D-20-00549

## Slide 10
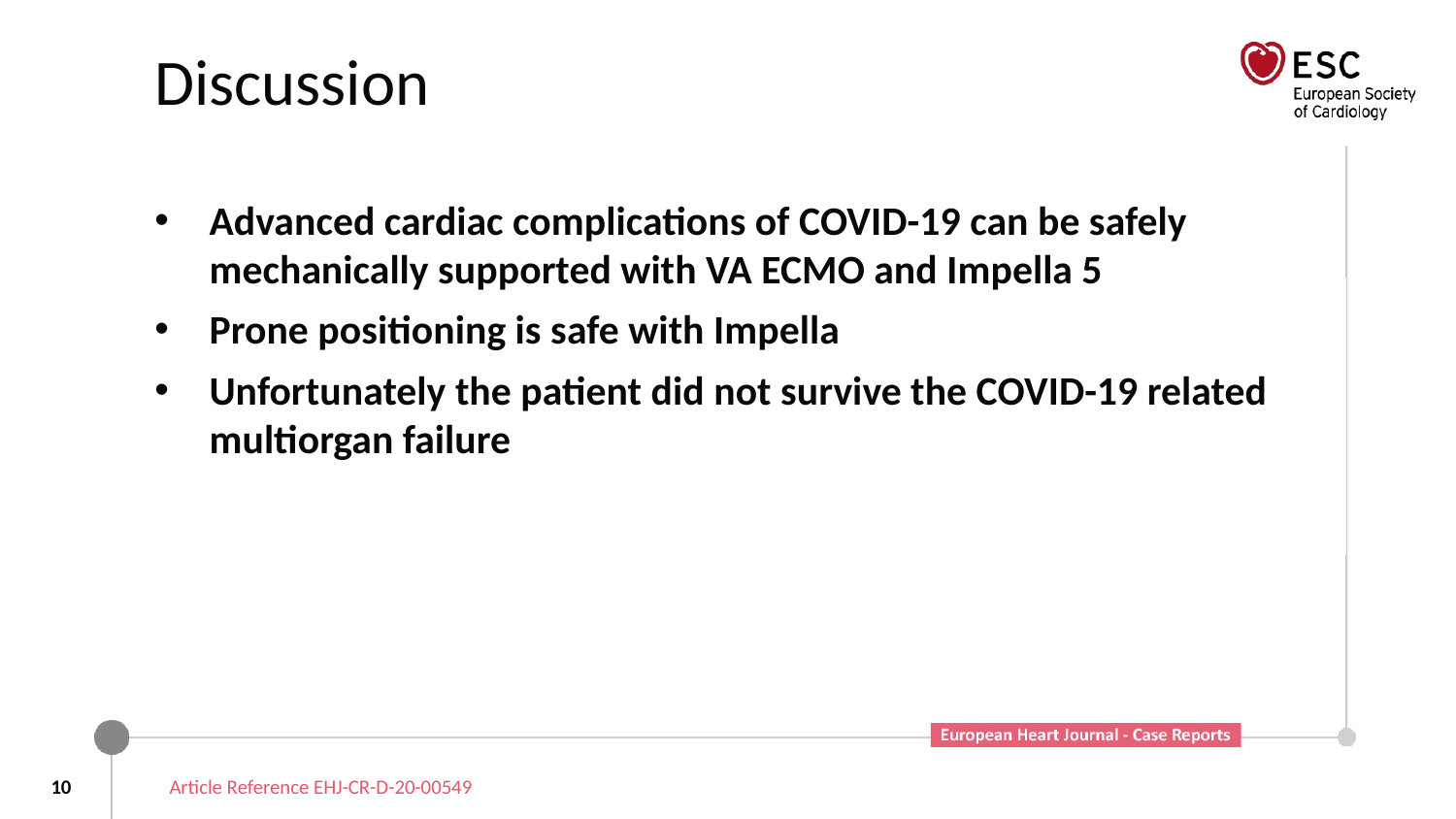

# Discussion
Advanced cardiac complications of COVID-19 can be safely mechanically supported with VA ECMO and Impella 5
Prone positioning is safe with Impella
Unfortunately the patient did not survive the COVID-19 related multiorgan failure
10
Article Reference EHJ-CR-D-20-00549

## Slide 11
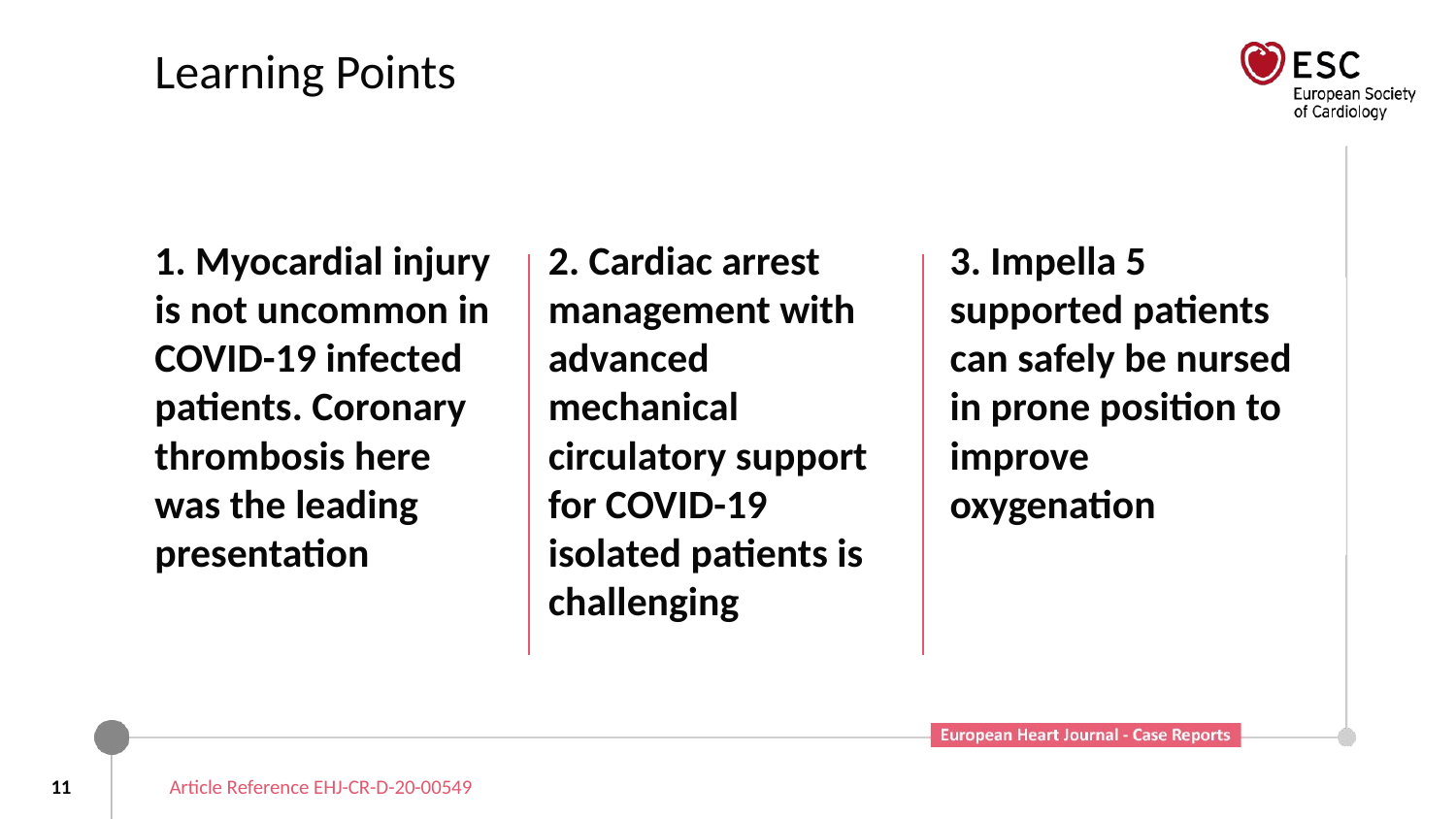

# Learning Points
1. Myocardial injury is not uncommon in COVID-19 infected patients. Coronary thrombosis here was the leading presentation
2. Cardiac arrest management with advanced mechanical circulatory support for COVID-19 isolated patients is challenging
3. Impella 5 supported patients can safely be nursed in prone position to improve oxygenation
11
Article Reference EHJ-CR-D-20-00549

## Slide 12
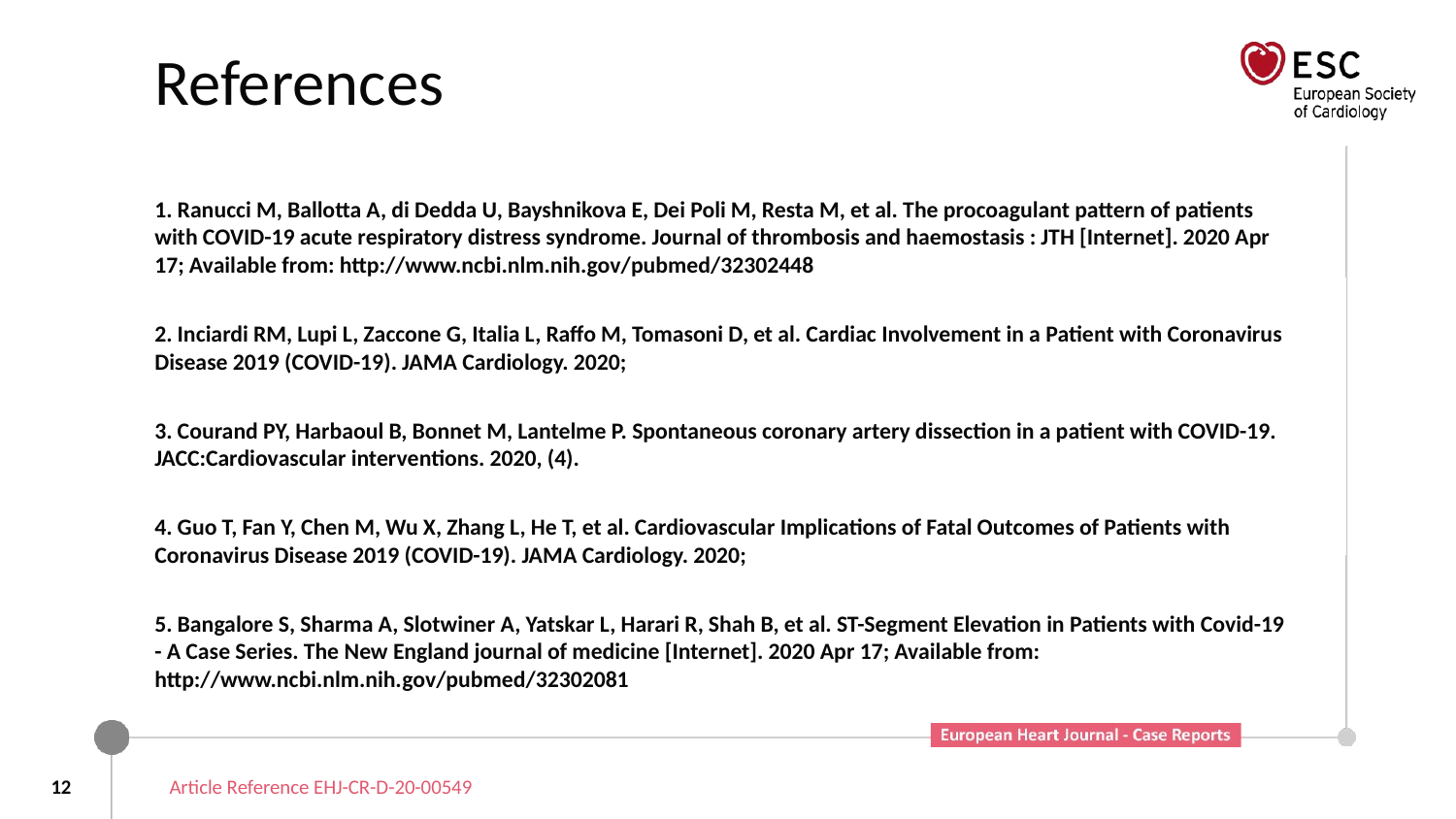

# References
1. Ranucci M, Ballotta A, di Dedda U, Bayshnikova E, Dei Poli M, Resta M, et al. The procoagulant pattern of patients with COVID-19 acute respiratory distress syndrome. Journal of thrombosis and haemostasis : JTH [Internet]. 2020 Apr 17; Available from: http://www.ncbi.nlm.nih.gov/pubmed/32302448
2. Inciardi RM, Lupi L, Zaccone G, Italia L, Raffo M, Tomasoni D, et al. Cardiac Involvement in a Patient with Coronavirus Disease 2019 (COVID-19). JAMA Cardiology. 2020;
3. Courand PY, Harbaoul B, Bonnet M, Lantelme P. Spontaneous coronary artery dissection in a patient with COVID-19. JACC:Cardiovascular interventions. 2020, (4).
4. Guo T, Fan Y, Chen M, Wu X, Zhang L, He T, et al. Cardiovascular Implications of Fatal Outcomes of Patients with Coronavirus Disease 2019 (COVID-19). JAMA Cardiology. 2020;
5. Bangalore S, Sharma A, Slotwiner A, Yatskar L, Harari R, Shah B, et al. ST-Segment Elevation in Patients with Covid-19 - A Case Series. The New England journal of medicine [Internet]. 2020 Apr 17; Available from: http://www.ncbi.nlm.nih.gov/pubmed/32302081
12
Article Reference EHJ-CR-D-20-00549
